# Complete genome of *Vibrio parahaemolyticus* FORC014 isolated from the toothfish

**DOI:** 10.1186/s13099-016-0134-0

**Published:** 2016-11-17

**Authors:** Sojin Ahn, Han Young Chung, Sooyeon Lim, Kwondo Kim, Suyeon Kim, Eun Jung Na, Kelsey Caetano-Anolles, Ju-Hoon Lee, Sangryeol Ryu, Sang Ho Choi, Heebal Kim

**Affiliations:** 1 Interdisciplinary Program in Bioinformatics, Seoul National University, Kwan-ak St. 599, Kwan-ak Gu, Seoul, 151-741 Republic of Korea; 2 Food-borne Pathogen Omics Research Center (FORC), Seoul National University, Seoul, Republic of Korea; 3 Department of Agricultural Biotechnology, Center for Food Safety and Toxicology, Seoul National University, Seoul, 08826 Republic of Korea; 4 Microbiomics and Immunity Research Center, Korean Research Institute of Bioscience and Biotechnology (KRIBB), 125 Gwahackro, Yuseong-gu, Daejeon, Republic of Korea; 5 C&K genomics, Seoul National University Research Park, Seoul, 151-919 Republic of Korea; 6 Department of Agricultural Biotechnology, Animal Biotechnology Major, and Research Institute of Agriculture and Life Sciences, Seoul National University, Seoul, 151-921 Republic of Korea; 7 Department of Food Science and Biotechnology, Kyung Hee University, 1732 Deokyoungdae-ro, Yongin, 17104 Republic of Korea

**Keywords:** *Vibrio parahaemolyticus*, Type III secretion system-2, Whole genome sequencing, Comparative genomics

## Abstract

**Background:**

Foodborne illness can occur due to various pathogenic bacteria such as *Staphylococcus aureus, Escherichia coli* and *Vibrio parahaemolyticus*, and can cause severe gastroenteritis symptoms. In this study, we completed the genome sequence of a foodborne pathogen *V. parahaemolyticus* FORC_014, which was isolated from suspected contaminated toothfish from South Korea. Additionally, we extended our knowledge of genomic characteristics of the FORC_014 strain through comparative analysis using the complete sequences of other *V. parahaemolyticus* strains whose complete genomes have previously been reported.

**Results:**

The complete genome sequence of *V. parahaemolyticus* FORC_014 was generated using the PacBio RS platform with single molecule, real-time (SMRT) sequencing. The FORC_014 strain consists of two circular chromosomes (3,241,330 bp for chromosome 1 and 1,997,247 bp for chromosome 2), one plasmid (51,383 bp), and one putative phage sequence (96,896 bp). The genome contains a total of 4274 putative protein coding sequences, 126 tRNA genes and 34 rRNA genes. Furthermore, we found 33 type III secretion system 1 (T3SS1) related proteins and 15 type III secretion system 2 (T3SS2) related proteins on chromosome 1. This is the first reported result of Type III secretion system 2 located on chromosome 1 of *V. parahaemolyticus* without thermostable direct hemolysin (*tdh*) and thermostable direct hemolysin-related hemolysin (*trh*).

**Conclusions:**

Through investigation of the complete genome sequence of *V. parahaemolyticus* FORC_014, which differs from previously reported strains, we revealed two type III secretion systems (T3SS1, T3SS2) located on chromosome 1 which do not include *tdh* and *trh* genes. We also identified several virulence factors carried by our strain, including iron uptake system, hemolysin and secretion system. This result suggests that the FORC_014 strain may be one pathogen responsible for foodborne illness outbreak. Our results provide significant genomic clues which will assist in future understanding of virulence at the genomic level and help distinguish between clinical and non-clinical isolates.

**Electronic supplementary material:**

The online version of this article (doi:10.1186/s13099-016-0134-0) contains supplementary material, which is available to authorized users.

## Background

*Vibrio parahaemolyticus* is an important gastrointestinal pathogen which is characterized by a gram-negative, rod shaped, and halophilic organism which causes food borne illness. When people eat oysters, shrimps, fish and other seafood contaminated with *V. parahaemolyticus,* they may develop a foodborne illness with serious gastroenteritis symptoms such as acute gastroenteritis, vomiting and even death [1].

The initial spread of *V. parahaemolyticus* caused an outbreak of foodborne illness in Japan in the early 1950s [2]. From that point on, food poisoning outbreaks caused by *V. parahaemolyticus* began to occur frequently worldwide [3]. With the goal of better understanding the spread of disease and prevention, numerous studies have been performed on *V. parahaemolyticus*, particularly focusing on how its toxins associate with food poisoning. While environmental strains rarely contain pathogenic genes thermostable direct hemolysin (*tdh*) and thermostable direct hemolysin-related hemolysin (*trh*), clinical strains which create foodborne illness, possess virulence factor including *tdh*, and *trh*. Therefore, *tdh*, and *trh* have been considered as the indicators of *V. parahaemolyticus* pathogenicity, which has an enterotoxic effect on the intestinal cells of the affected mammal [4, 5]. Recent studies, however, announced that some clinical strains identified negative for *tdh* and *trh* genes [4, 5]. In addition to the two previously mentioned pathogenicity indicators, T3SS2, which is required for intestinal colonization, has been speculated to be a possible indicator of *V. parahaemolyticus* pathogenicity [5–8]. However, major virulence indicators of *V. parahaemolyticus* at the genomic level are still unclear despite the many studies which have been performed which attempted to identify them.

In this study, we sequenced the putative clinical strain *V. parahaemolyticus* FORC_014, which was isolated from toothfish which was suspected to have caused a spread of foodborne illness in South Korea. The whole genome sequences of *V. parahaemolyticus* will help to understand genetic variation between non-pathogenic strain and pathogenic strains. In addition, we performed comparative analysis on the FORC_014 strain with eight other complete genome sequences from public databases to gain genomic level information and greater understanding of this strain.

## Methods

### Genomic DNA preparation and whole genome sequencing

*Vibrio parahaemolyticus* FORC_014, a strain of *V. parahaemolyticus* which was isolated from contaminated fried toothfish in Busan, South Korea, was received from the Ministry of Food and Drug Safety. Total genomic DNA preparation was performed using a Qiagen blood and tissue kit following manufacturer’s protocol.

Approximately 5 μg of DNA was fragmented to 8–12 kbp using the Hydroshear system and assembly of DNA was performed at a shearing speed of 9 for 20 cycles. PacBio DNA Template Prep Kit 2.0 (3–10 kbps), used for SMRT Sequencing with C2 chemistry on PacBio RS, was used for SMRTbell library preparation following manufacturer’s instructions. The size distribution of the purified DNA template was measured using an Agilent 12,000 DNA kit and the concentration of the template was measured using Invitrogen Qubit. Primers were annealed to the template and DNA polymerase C2 was added following the manufacturer’s recommendations. Enzyme-template complexes were set up with DNA/Polymerase Binding Kit P4 (PacBio) on the 75,000 zero-mode waveguides (ZMWs). DNA sequencing Reagent 2.0 kit (Pacific Bioscience) was used for SMRTbell library sequencing with a long (1 × 120 min) sequence capture protocol for maximizing read length with PacBio RS II. The summary of sequencing result is included Additional file 1.

### Genome assembly and annotation

Sequencing reads were assembled within the SMRT portal system [9]. The whole genome was assembled using HGAP assembly version 3 algorithm with curation of genome size parameter which was set to 5,100,000 bp. The more statistics information from HGAP assembly is provided Additional file 1. Re-sequencing and variant polishing was performed on contigs which were generated after first draft assembly to resolve the problem of high error using the PacBio RS II sequencing system. Determination of orientation and the direction of assembled sequence was performed using the Basic Local Alignment Search Tool (BLAST) and MUMmer analysis by comparison with the reference genome, *V. parahaemolyticus* CDC_K4557 [10]. The polished sequence was manually curated using Bioedit software [11].

Rapid Annotation of Prokaryotic Genomes(PROKKA), which includes prediction tools such as Prodigal [12], RNAmmer [13], Aragorn [14], SignalP [15], and infernal [16], was used for Open Reading Frame, tRNA and rRNA prediction of *V. parahaemolyticus* FORC_014 [17]. We also used Rapid Annotation through the Subsystem Technology server in order to confirm ORFs [18]. After gene prediction, we characterized gene function based on Cluster of Orthologous Groups (COG) annotation using the Web server for fast Metagenomic Sequence Analysis (WebMGA) with default options and for subsystem functional categorization [19], SEED annotation was performed using the SEED viewer within the RAST server. Sequences of virulence factors from the in Virulence Factor Database (VFDB; www.mgc.ac.cn/VFs/) were used for defining virulence factors in all strains, except for the well-defined strain RIMD2210633, using BLASTn method (identity ≥0.90; query coverage ≥0.90).

### Comparative genome analysis

In this study, the complete genome sequences of eight *V. parahaemolyticus* strains: RIMD2210633, CDC_K4557, BB22OP, FORC_008, UCM-V493, FORC_006, FORC_004, and FDA_R31 were downloaded from NCBI (http://www.ncbi.nlm.nih.gov/genome/genomes/691) and used for comparative analysis.

For calculation of the Average Nucleotide identity (ANI) value among 9 strains, the Jspecies tool based on the BLAST algorithm was used [20]. Each of query genome was cut into small fragments of 1020 bp and high scoring pairs between two sequences were selected using the BLAST algorithm for calculating ANI values [21]. After that, a genome tree was constructed using the unweighted pair group method in R software. After selection of the genome using ANI values, comparison of the genome sequence was performed using the Artemis comparison tool (ACT) and confirmed unmatched regions [22].

Also, BLAST search was used to predict virulence factors of FORC_014 strain. The Virulence Factors Database (www.mgc.ac.cn/VFs/) was used as subject sequence database and FORC_14 strain sequence used as query sequence.

## Quality assurance

The 16 s rRNA gene of *V. parahaemolyticus* FORC_014 was isolated from the completely assembled sequence using RNAmmer within the PROKKA annotation tool. The complete genome sequence of the same species was used to calculate the distance through comparison of ANI values.

## Initial findings

### Genome properties

**Fig. 1:**
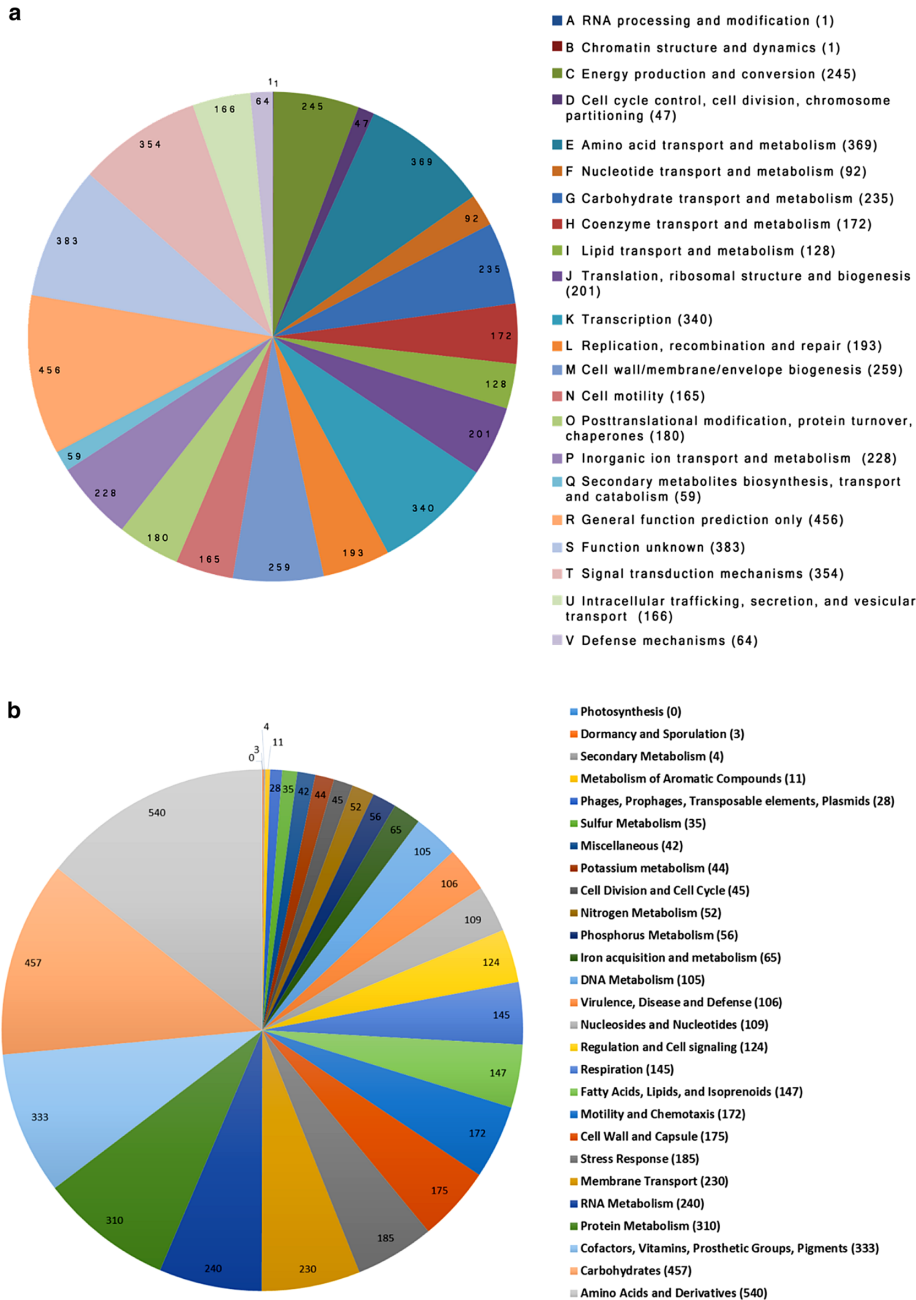
Functional categorization of FORC_014 based on (**a**) the COG database and (**b**) the SEED database

The complete genome of *V. parahaemolyticus* FORC_014 includes two circular DNA chromosomes of 3,241,330 and 1,997,247 bp with GC contents of 45.2–45.7%, one plasmid of 51,383 bp with a GC content of 40.9% and a phage of 96,896 bp with a GC contents of 46.7%. The strain FORC_014 chromosomes contained a total of 4274 putative protein coding sequences and 160 RNA genes. More information about the FORC_014 genome is given in Table 1. The predicted open reading frames (ORFs) were categorized into COG functional groups. The result of COG categorization is shown in Fig. 1a. Among the COG analysis result, class of R (456 ORFs, general function prediction only), class of S (354 ORFs, Function unknown), class of E (369 ORFs, Amino acid transport and metabolism), class of T (354 ORFs, Signal transduction mechanisms), and class of K (340 ORFs, Transcription) were abundant groups based on count. For categorization of subsystem features, we performed SEED subsystem categories analysis (Fig. 1b). As a result of the SEED analysis, 3763 ORFs were classified to SEED subsystem categories. Among the SEED categorization, Amino Acids and Derivatives (540 ORFs), Carbohydrates (457 ORFs), Cofactors, Vitamins, Prosthetic Groups, Pigments (333 ORFs), Protein Metabolism (310 ORFs) and RNA Metabolism (240 ORFs) were abundant categories.

**Table 1:** Genomic features of *V.parahaemolyticus* FORC_ 014

Gene feature	Chromosome 1	Chromosome 2	Plasmid	Phage
Genome size (bp)	3,241,330	1,997,247	51,383	96,896
GC contents (%)	45.2	45.7	40.9	46.7
Open reading frames	2944	1782	54	133
Annotated genes	2212	1340	31	23
Hypothetical genes	732	442	23	110
tRNAs	96	30	0	0
rRNAs	25	9	0	0
Accession number	CP011406	CP011407	CP011408	–

## Results and discussion

Genome tree analysis was performed on 8 complete genomes of *V. parahaemolyticus* strains gathered from the NCBI database. Average nucleotide identity values (ANI) were calculated with these 8 strains and a dendrogram was constructed using ANI values. All of values among strains are higher than 95% identity which known as criteria of the same species. As a result, the FORC_014 strain was found to be clustered with FORC_006 and UCM_V493 strain. The FORC_006 strain was isolated from South Korea and UCM_V493 strain was environmentally isolated in Spain [23]. This comparison data is shown as a dendrogram and table in Additional file 2. We notice that our strain scored slightly higher with UCM_V493 strain than other clinical strains.

**Fig. 2:**
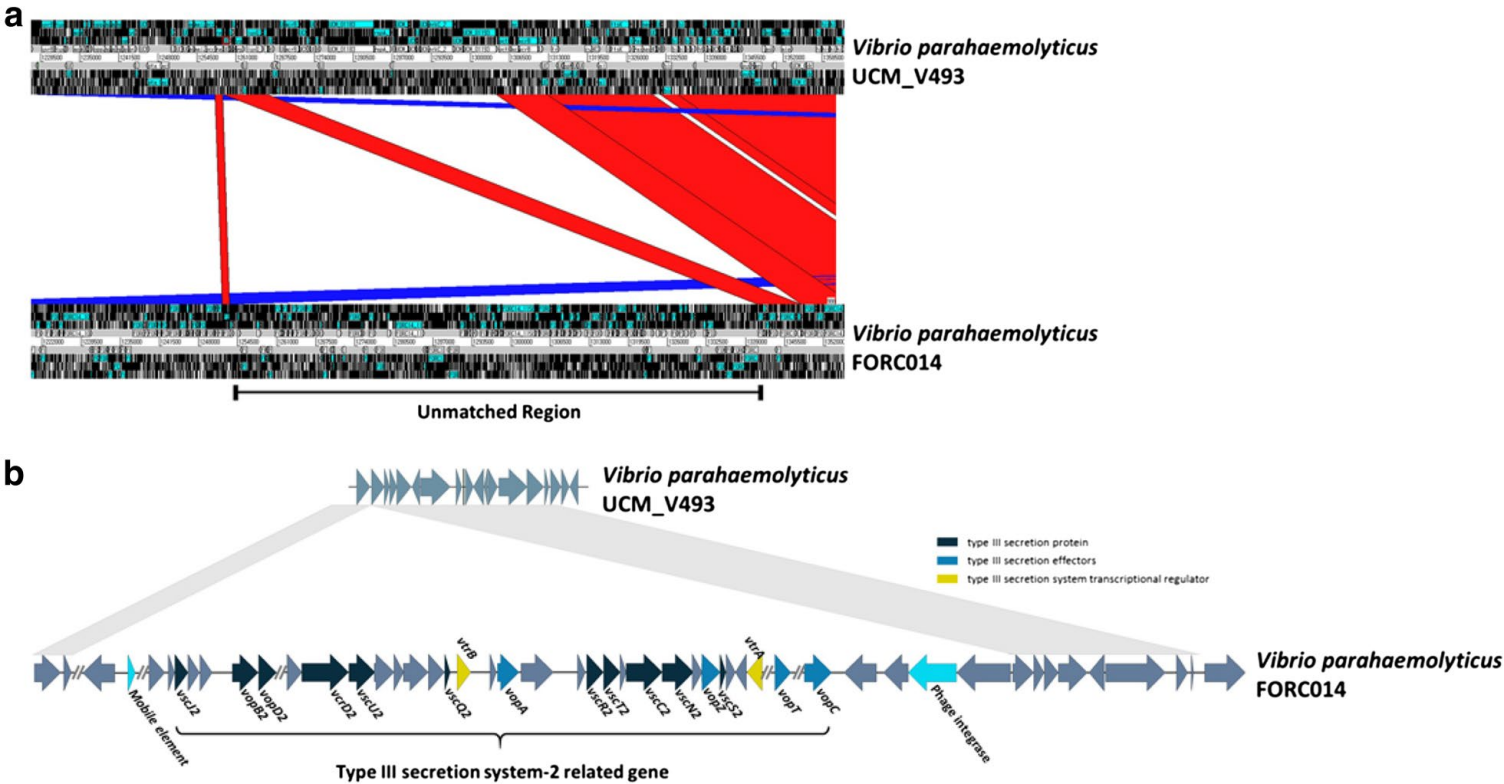
Comparative genome map between UCM-V493 chromosome 1 and FORC_014 chromosome 1. **a** Visualization of unmatched regions between UCM-V493 and FORC_014 using the Artemis Comparison Tool. **b** Gene map of unmatched regions. The unmatched regions of FORC_014 containing Type III secretion system-2 related genes and several mobile elements

In addition, we performed a comparison with the UCM_V493 sequence to determine the difference between the two strains using ACT. From the comparison, we identified a noticeable unmatched region on chromosome 1 (1,253,195–1,341,058 for FORC_014). This region of FORC_014 contains Type III secretion system2 (T3SS2) proteins (Fig. 2). Interestingly, previous studies described Type III secretion system1 (T3SS1) genes located on chromosome 1 and T3SS2 genes located on chromosome 2 in *V. parahaemolyticus* [24]. However, our strain contained both T3SS1 (1,937,875–1,975,436 region) and T3SS2 genes on chromosome 1, which has not been reported to date (Additional file 3). In order to verify our identification of T3SS2 genes on chromosome 1 of FORC_014, we compared another typical *V. parahaemolyticus* strain, RIMD2210633, using ACT. Moreover, we defined T3SS1 and T3SS2 genes in our strain using the BLAST method, which produced the same result. T3SS2 has been described as a major essential factor for enterotoxicity and intestinal colonization [8, 25]. Particularly, the *vopB2*(FORC14_1152) gene was detected in this T3SS2 region on chromosome 1. Previous studies have suggested the *vopB2* gene as a possible indicator of strain virulence substitute for *tdh* or *trh* [26]. Additionally, we found mobile elements (1,279,403–1,279,702 region) and phage integrase (1,336,825–1,338,777 region) in unmatched regions near the T3SS2. Considering these overall results, these mobile elements might be involved in translocation of these gene clusters, including T3SS2. This result suggests that T3SS2 may play a role in the pathogenesis of FORC_014.

Our results also revealed that the FORC_014 strain does not encode *tdh* and *trh* genes, which are known to be major virulence factors of *V. parahaemolyticus.* However, we detected that FORC_014 strain encoded various virulence factors including two type 3 secretion systems (T3SSs) using the BLAST method (Additional file 4). FORC_014 contains various iron uptake-associated genes (Enterobactin receptors; *irgA,* and *vdtA*, Periplasmic binding protein-dependent ABC transport systems; *vctP, vctD, vctG,* and *vctC,* Heme receptors; *hutA*, and *hutR*, vibrioferrin associated; *pvuA,B,C,D,E*, *pvsA,B,C,D,E,* and *psuA)*, and hemolysin (tlh;FORC14_3316). Additionally, we performed LDH release assay using the INT-407 cells for testing cytotoxicity activity (Additional file 5). The test result supported that FORC_014 strain has pathogenesis activity. Based on these results, we suggest that FORC_014 is pathogenic, even though it is *tdh* and *trh* negative [5, 6, 27, 28].

In conclusion, we completed genomic sequencing of *V. parahaemolyticus* FORC_014, which is considered a leading cause of foodborne illness from comparative studies with already published strains. As a result, we found pathogenic island regions of FORC_014 that clustered T3SS1 related genes and T3SS2 related genes on chromosome 1. Our findings provide not only new information about virulence related genes, especially T3SS2 on Chromosome 1 of *V. parahaemolyticus,* but also could support results of previous studies on the pathogenicity of *tdh* and *trh* negative clinical strains. Further comparative genome studies of clinical and environmental isolates with our *V. parahaemolyticus* strain will provide information crucial to revealing the major pathogenic mechanism.

## Additional files

**Additional file 1.** Summary of *V. parahaemolyticus* FORC_014 genome.

**Additional file 2.** Distance dendrogram among *Vibrio parahaemolyticus* strains based on ANI values. The dendrogram indicated that FORC_014 closely related with UCM-V493 based on ANI value.

**Additional file 3.** List of Type III secretion system-2 related genes in the complete genome of *V. parahaemolyticus* strains.

**Additional file 4.** Virulence factors of *V. parahaemolyticus* FORC_014.

**Additional file 5.** Cytotoxicity analysis for two strains of *V. parahaemolyticus*. INT-407 cells were infected with *V. parahaemolyticus* FORC_014 and KCTC2471 (*tdh* positive, and *trh* negative strain) as control at two levels of multiplicity of infection (MOIs) for (A) 2 h and (B) 3 h. The cytotoxicity of these strains was expressed using the total LDH release of the completely lysed cells, measured by LDH release assay. Error bars represent the standard error of the mean (SEM).

## Abbreviations

FORC: Food-borne Pathogen Omics Research Center
KRIBB: Korean Research Institute of Bioscience and Biotechnology
T3SS1: type III secretion system 1
T3SS2: type III secretion system 2
tdh: thermostable direct hemolysin
trh: thermostable direct hemolysin-related hemolysin
ACT: artemis comparison tool
ANI: average nucleotide identity
ORF: open reading frame
COG: Cluster of Orthologous Groups
WebMGA: Web server for fast Metagenomic Sequence Analysis
BLAST: Basic Local Alignment Search Tool
LDH: lactate dehydrogenase
