# Supplementary material for: Complete genome of Vibrio parahaemolyticus FORC014 isolated from the toothfish
Source: Gut Pathog. 2016 Nov 17;8:59. doi: 10.1186/s13099-016-0134-0 (PMC5114773; doi:10.1186/s13099-016-0134-0)
Supplement: Supplementary file 1 — Additional file 1. Summary of V. parahaemolyticus FORC_014 genome. [file 13099_2016_134_MOESM1_ESM.docx]

**Materials and Methods**

**Cytotoxicity test**

Cytotoxicity test of FORC_014 strain was conducted by measuring cytoplasmic lactate dehydrogenase (LDH) activity that is observed from the human epithelial INT-407 cells which were completely lysed by 2% Triton X 100. The INT-407 cells were grown in minimum essential medium containing 1% (v/v) fetal bovine serum (MEMF) (Gibco-BRL, Gaithersburg, MD) in 96-well culture dishes (Nunc, Roskilde, Denmark) as described previously [1]. Each well of the INT-407 2 × 10^4^ cells was infected with *V. parahaemolyticus* FORC_014 and KCTC 2471 as a control at various multiplicities of infection (MOIs) for 2hours or 3hours. The LDH- releasing activity into the supernatant was determined using a cytotoxicity detection kit (Roche, Mannheim, Germany).

**References**

1. Kim S, Bang Y-J, Kim D, et al. Distinct characteristics of OxyR2, a new OxyR-type regulator, ensuring expression of Peroxiredoxin 2 detoxifying low levels of hydrogen peroxide in *Vibrio vulnificus*. Mol microbiol. 2014;93:992-1009.

**Additional file 1. Summary of *V. parahaemolyticus* FORC_014 genome.**

| **Property** | **Term** |
| --- | --- |
| Finishing quality | Finished |
| Libraries used | PacBio SMRTbell™ library |
| Number of SMRT cells | 5 |
| Sequencing platforms | PacBio RS II sequencer |
| Assemblers | PacBio SMRT analysis 3.0 |
| Gene calling method | PROKKA |
| Number of reads | 114,315 (PacBio_20K) |
| N50 read length | 7,967 |
| Average genome coverage | 92.91x |
| Contigs no. | 4 |
| Scaffolds no. | 4 |
| N50 contig length | 3,241,333 |
| Chromosome length (bp) | 3,241,330 (Chromosome I) 1,997,247 (Chromosome II) 51,383 (Plasmid) 96,896 (Phage) |
| Locus Tag | FORC14 |
| Genbank ID | CP011406, CP011407, CP011408 |
| BIOPROJECT | PRJNA280138 |
| Source Material Identifier | FORC_014 |
