# Supplementary material for: Complete genome of Vibrio parahaemolyticus FORC014 isolated from the toothfish
Source: Gut Pathog. 2016 Nov 17;8:59. doi: 10.1186/s13099-016-0134-0 (PMC5114773; doi:10.1186/s13099-016-0134-0)

**Additional file 2.** **Distance dendrogram among *Vibrio parahaemolyticus* strains based on ANI values**. The dendrogram indicated that FORC_014 closely related with UCM-V493 based on ANI value.


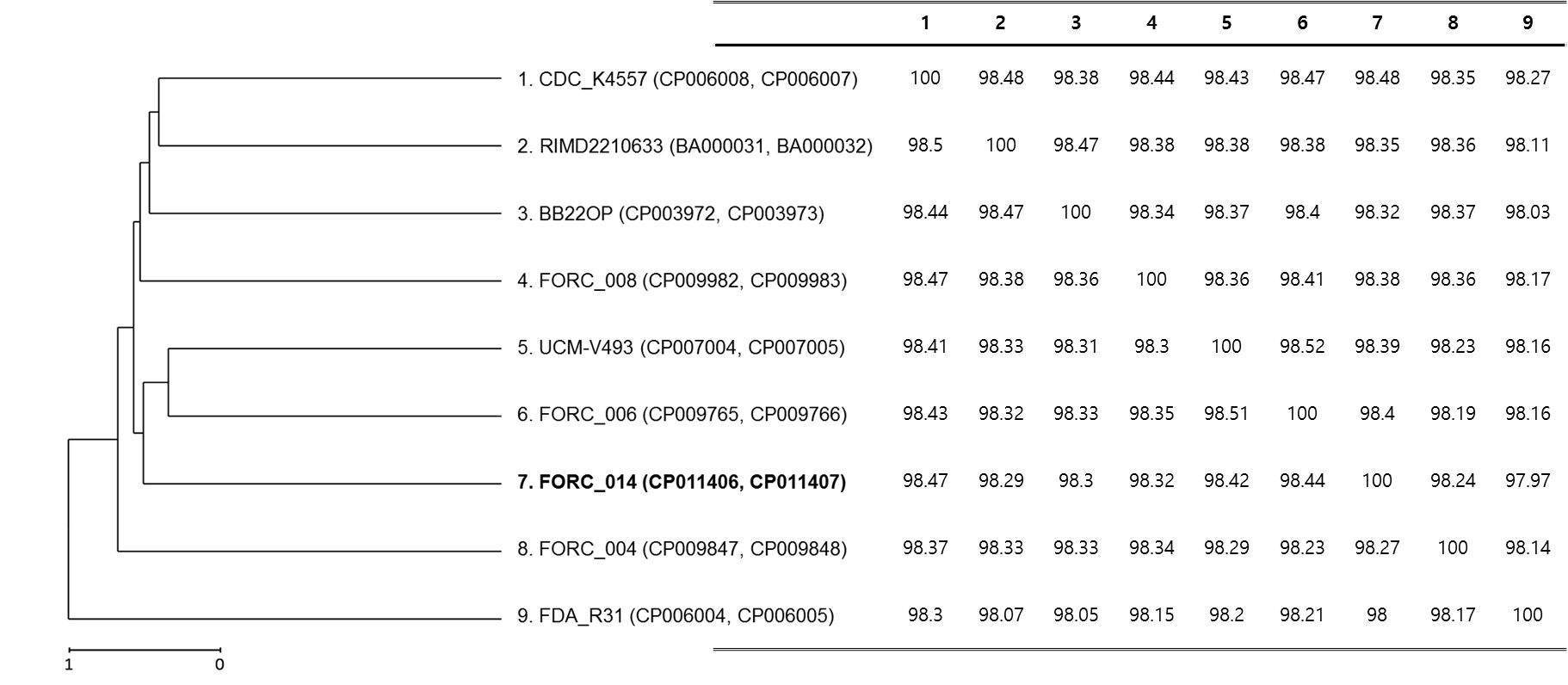

Supplement: Supplementary file 2 — Additional file 2. Distance dendrogram among Vibrio parahaemolyticus strains based on ANI values. The dendrogram indicated that FORC_014 closely related with UCM-V493 based on ANI value. [file 13099_2016_134_MOESM2_ESM.docx]
