# Supplementary material for: Complete genome of Vibrio parahaemolyticus FORC014 isolated from the toothfish
Source: Gut Pathog. 2016 Nov 17;8:59. doi: 10.1186/s13099-016-0134-0 (PMC5114773; doi:10.1186/s13099-016-0134-0)
Supplement: Supplementary file 3 — Additional file 3. List of Type III secretion system-2 related genes in the complete genome of V. parahaemolyticus strains. [file 13099_2016_134_MOESM3_ESM.docx]

**Additional file 3. List of Type III secretion system-2 related genes in the complete genome of *V.parahaemolyticus* strains**

|  |  | **RIMD2210633** | **CDC_K4557** | **BB22OP** | **FORC_008** | **UCM-V493** | **FORC_014** | **FDA_R31** |
| --- | --- | --- | --- | --- | --- | --- | --- | --- |
|  |  | tdh+ trh+ | tdh− trh− | tdh+ trh- | tdh− trh− | tdh− trh− | tdh− trh− | tdh+ trh+ |
| **T3SS2** | | | | | | | | |
|  | **OspC2** | VPA1331 | - | VPBB_A1210 (putative) | - | - | - | - |
|  | **VscS2** | VPA1335 | - | VPBB_A1213 | - | - | FORC14_1179 | - |
|  | **vscN2** | VPA1338 | - | VPBB_A1216 | - | - | FORC14_1176 | - |
|  | **vscC2** | VPA1339 | - | VPBB_A1217 | - | - | FORC14_1175 | - |
|  | **vscU2** | VPA1354 |  | VPBB_A1231 |  |  | FORC14_1161 | - |
|  | **vcrD2** | VPA1355 | - | VPBB_A1232 | - | - | FORC14_1160 | - |
|  | **VscT2** | VPA1341 | - | VPBB_A1219 | - | - | FORC14_1173 | - |
|  | **VscR2** | VPA1342 | - | VPBB_A1220 | - | - | FORC14_1172 | - |
|  | **VscQ2** | VPA1349 | - | VPBB_A1226 | - | - | FORC14_1166 | - |
|  | **VopD2** | VPA1361 |  |  |  |  | FORC14_1153 | - |
|  | **VopB2** | VPA1362 | - | VPBB_A1239 | - | - | FORC14_1152 | - |
|  | **VscJ2** | VPA1367 | - | VPBB_A1245 | - | - | FORC14_1149 | - |
|  | **OspB** | VPA1380 | - | VPBB_A1257 | - | - | - | - |
| **T3SS2 secreted effectors** | | | | | | | | |
|  | **VopA** | VPA1346 |  | VPBB_A1223 |  |  | FORC14_1169 | - |
|  | **VopT** | VPA1327 |  | VPBB_A1207 |  |  | FORC14_1187 | - |
|  | **VopC** | VPA1321 |  | VPBB_A1202 |  |  | FORC14_1193 | - |
|  | **VopL** | VPA1370 |  | VPBB_A1249 |  |  | FORC14_1146 | - |
