# Supplementary material for: Complete genome of Vibrio parahaemolyticus FORC014 isolated from the toothfish
Source: Gut Pathog. 2016 Nov 17;8:59. doi: 10.1186/s13099-016-0134-0 (PMC5114773; doi:10.1186/s13099-016-0134-0)
Supplement: Supplementary file 4 — Additional file 4. Virulence factors of V. parahaemolyticus FORC_014. [file 13099_2016_134_MOESM4_ESM.docx]

**Additional file 4. Virulence factors of *V. parahaemolyticus* FORC_014**

| Virulence factor | Annotation | Chromosome | Location (locus_tag) |
| --- | --- | --- | --- |
| Adherence | | | |
| *mshH, mshI, mshJ, mshK, mshL,*  *mshM, mshN, mshE, mshG, mshF, mshA* | Mannose-sensitive hemagglutinin (MSHA type IV pilus) | Chromosome 1 | 3088545~3101090 (FORC14_2819, FORC14_2821~FOR14_2830) |
| *pilB, pilC, pilD* | Type IV pilus | Chromosome 1 | 2895010~2898879 (FORC14_2644~FORC14_2646) |
| Antiphagocytosis | | | |
| *cpsA, cpsB, cpsC, cpsE, cpsF, cpsG, cpsH, cpsI, cpsJ* | Capsular polysaccharide | Chromosome 2 | 1562593~ 1574187 (FORC14_4360~FORC14_4362, FORC14_4364~FOR14_4369) |
| *flgB, flgC, flgD, flgE, flgF, flgG, flgH, flgI, flgJ, flgK, flgL, flgN, flgM, flgA,* | flagella | Chromosome 1 Chromosome 2 | 900432~914763  (FORC14_0796~FORC14_0806) 452879~ 461799  (FORC14_3352~FORC14_3362) 896634~ 898277  (FORC14_0791~FORC14_0793) 451125~ 452800  (FORC14_3349~FORC14_3351) |
| *cheV, cheR,* | Chemotaxis protein | Chromosome 1 | 898354~ 900119  (FORC14_0794~FORC14_0795) |
| *fliA, flhG, flhF,  fliL, fliK,  flrC, flrB, flrA, fliS, flaI, fliD, flaG, flaB, flaC, flaE* | flagella | Chromosome 1 | 2574910~2578020  (FORC14_2358~FORC14_2360) 2585422~2587906  (FORC14_2369~FORC14_2370) 2593968~2605595  (FORC14_2377~FORC14_2387) |
| *flhA, flhB, fliR, fliQ, fliP, fliO, fliN, filM, fliJ, fliI, fliH, fliG, fliF, fliE,* | flagella | Chromosome 1 Chromosome 2 | 2578079~ 2585414  (FORC14_2361~FORC14_2368) 1758832~ 1765099  (FORC14_4516~FORC14_4534) 2588081~2593841  (FORC14_2371~FORC14_2376) 1750421~1756074  (FORC14_4508~FORC14_4513) |
| *cheW, cheB, cheA, cheZ, cheY* | Chemotaxis protein | Chromosome 1 | 2567785~2574876  (FORC14_2351, FORC14_2354~ FORC14_2357) |
| *motA, motB motY, motX* | Flagellar motor protein | Chromosome 1 Chromosome 2 | 810932~812656  (FORC14_0717~FORC14_0718) 1773819~1775668  (FORC14_4534~FORC14_4535) 2464862~2465743  (FORC14_2257) 1757422~1758444  (FORC14_4515) 224120~224755  (FORC14_3149) |
| Iron uptake | | | |
| *irgA, vctA* | Enterobactin receptors | Chromosome 1 Chromosome 2 | 2976278~2978236  (FORC14_2719) 820699~822705  (FORC14_3696) |
| *vctP, vctD, vctG, vctC* | Periplasmic binding protein-dependent ABC transport systems | Chromosome 2 | 815388~818955  (FORC14_3689~FORC14_3692) |
| *hutA, hutR* | Heme receptors | Chromosome 2 | 1082440~1084521  (FORC14_3907) 1650624~1652762  (FORC14_4424) |
| *pvuE, pvuD, pvuC, pvuB* | vibrioferrin utilization | Chromosome 2 | 1885158~1888850  (FORC14_4626~FORC14_4629) |
| *pvuA* | ferric vibrioferrin receptor | Chromosome 2 | 1888952~1891090  (FORC14_4630) |
| *pvsA, pvsB, pvsC, pvsD, pvsE* | vibrioferrin synthesis | Chromosome 2 | 1893497~1900745  (FORC14_4632~FORC14_4636) |
| *psuA* | Iron acquisition systems | Chromosome 2 | 1891213~1893249  (FORC14_4631) |
| Quorum sensing | | | |
| *luxS* | Autoinducer-2 | Chromosome 1 | 2907009~2907527  (FORC14_2657) |
| *cqsA* | Cholerae autoinducer-1 | Chromosome 2 | 893759~894940  (FORC14_3741) |
| Secretion system | | | |
| *epsC, epsE, epsF, epsG, epsH,, epsJ, epsK, epsL, epsM, epsN* | EPS type II secretion system | Chromosome 1 | 259047~270364  (FORC14_0215, FORC14_0217~FORC14_0220, FORC14_0222~FORC14_0226) |
| *vopD, vopB, vcrH, vcrV, vcrG, vcrR, vcrD, vscY, vscX, sycN, tyeA, vopN, vscN, vscO, vscP, vscQ, vscR, vscS, vscT, vscU, vecA, vscL, vscK, vscJ, vscI, vscH, vscG, vscF, vscD, vscC, vscB, exsD, virG, vxsC* | type III secretion protein (TTSS-1) | Chromosome 1 | 1937875~ 1954593  (FORC14_1727~ FORC14_1746) 1959949~1960407  (FORC14_1752) 1964122~1971559  (FORC14_1757~FORC14_1763, FORC14_1765~ FORC14_1767) 1971572~1975436  (FORC14_1768, FORC14_1770~FORC14_1771) |
| *vopQ, vopR, vopS* | TTSS-1 secreted effectors | Chromosome 1 | 1958461~1959939  (FORC14_1751) 1960634~1961611  (FORC14_1753) 1962314~1963477  (FORC14_1755) |
| *vscJ2, vopB2, vopD2 vcrD2*, *vscU2 vscQ2, vtrB, vscR2, vscT2, vscC2, vscN2, vscS2, vtrA* | type III secretion protein (TTSS-2) | Chromosome 1 | 1292557~ 1296666  (FORC14_1149, FORC14_1152~FORC14_1153) 1304596~ 1307555  (FORC14_1160~FORC14_1161) 131038~ 1311433  (FORC14_1166~FORC14_1167) 1316124~1320440  (FORC14_1172~FORC14_1173, FORC14_1175~FORC14_1176) 1321515~1321748  (FORC14_1179) 1322607~1323221  (FORC14_1182) |
| *vopA/vopP, vopZ, vopT, vopC* | TTSS-2 secreted effectors | Chromosome 1 | 1312515~1313384  (FORC14_1169) 1320782~1321528  (FORC14_1178) 1327903~1328541  (FORC14_1187) 1332621~1333727  (FORC14_1193) |
| Toxin | | | |
| *tlh* | Thermolabile hemolysin | Chromosome 2 | 416057~417310  (FORC14_3316) |
