# Supplementary material for: Complete genome of Vibrio parahaemolyticus FORC014 isolated from the toothfish
Source: Gut Pathog. 2016 Nov 17;8:59. doi: 10.1186/s13099-016-0134-0 (PMC5114773; doi:10.1186/s13099-016-0134-0)
Supplement: Supplementary file 5 — Additional file 5. Cytotoxicity analysis for two strains of V. parahaemolyticus. INT-407 cells were infected with V. parahaemolyticus FORC_014 and KCTC2471 (tdh positive, and trh negative strain) as control at two levels of multiplicity of infection (MOIs) for (A) 2 h and (B) 3 h. The cytotoxicity of these strains was expressed using the total LDH release of the completely lysed cells, measured by LDH release assay. Error bars represent the standard error of the mean (SEM). [file 13099_2016_134_MOESM5_ESM.docx]

**Additional file 5**

**
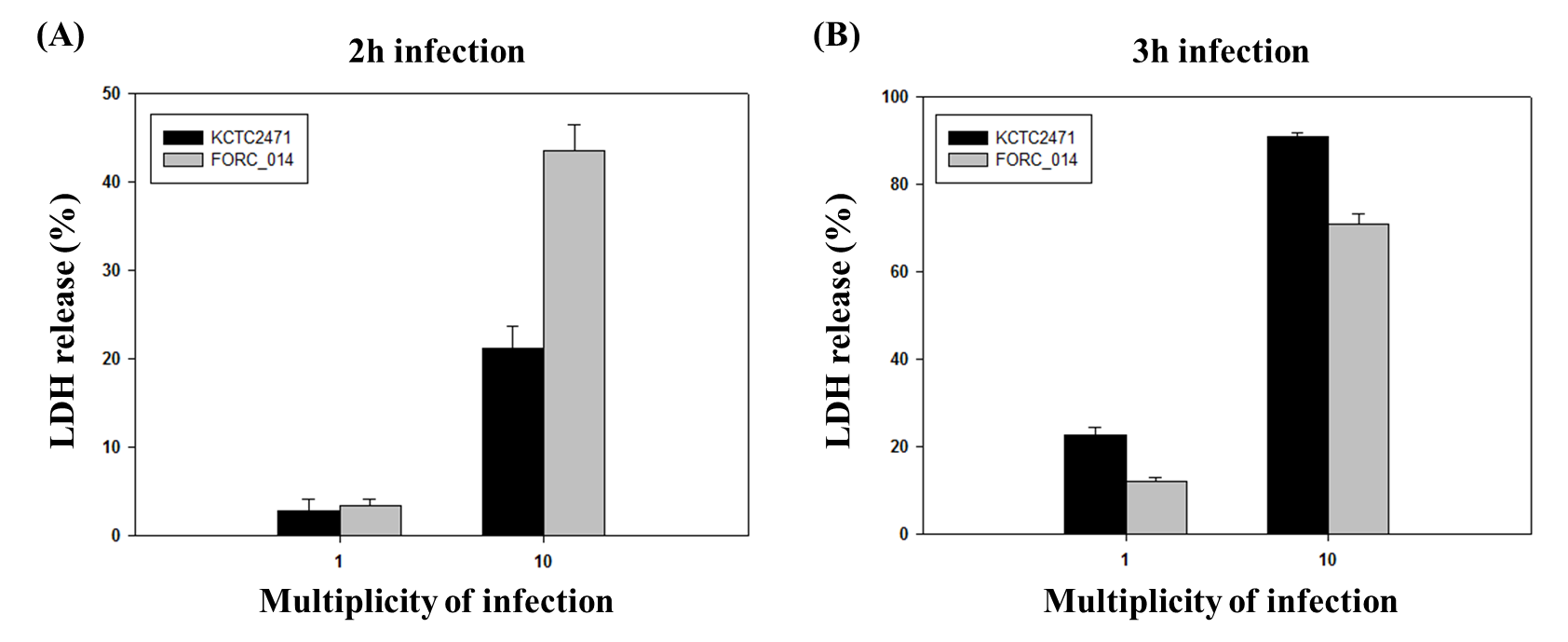
**

**Additional file 5. Cytotoxicity analysis for two strains of *V. parahaemolyticus.*** INT-407 cells were infected with *V. parahaemolyticus* FORC_014 and KCTC2471 (*tdh* positive, and *trh* negative strain) as control at two levels of multiplicity of infection (MOIs) for (A) 2h and (B) 3h. The cytotoxicity of these strains was expressed using the total LDH release of the completely lysed cells, measured by LDH release assay. Error bars represent the standard error of the mean (SEM).
